# Terminal Mono- and Bis-Conjugates of Oligonucleotides with *Closo*-Dodecaborate: Synthesis and Physico-Chemical Properties

**DOI:** 10.3390/ijms22010182

**Published:** 2020-12-26

**Authors:** Darya S. Novopashina, Mariya A. Vorobyeva, Alexander A. Lomzov, Vladimir N. Silnikov, Alya G. Venyaminova

**Affiliations:** Institute of Chemical Biology and Fundamental Medicine SB RAS, Lavrentiev Ave. 8, 630090 Novosibirsk, Russia; maria.vorobjeva@gmail.com (M.A.V.); lomzov@niboch.nsc.ru (A.A.L.); silnik@niboch.nsc.ru (V.N.S.); ven@niboch.nsc.ru (A.G.V.)

**Keywords:** boron clusters, *closo*-dodecaborate, oligonucleotide conjugates, click-chemistry, duplex stability, duplex structure

## Abstract

Oligonucleotide conjugates with boron clusters have found applications in different fields of molecular biology, biotechnology, and biomedicine as potential agents for boron neutron capture therapy, siRNA components, and antisense agents. Particularly, the *closo*-dodecaborate anion represents a high-boron-containing residue with remarkable chemical stability and low toxicity, and is suitable for the engineering of different constructs for biomedicine and molecular biology. In the present work, we synthesized novel oligonucleotide conjugates of *closo*-dodecaborate attached to the 5′-, 3′-, or both terminal positions of DNA, RNA, 2′-O-Me RNA, and 2′-F-Py RNA oligomers. For their synthesis, we employed click reaction with the azido derivative of *closo*-dodecaborate. The key physicochemical characteristics of the conjugates have been investigated using high-performance liquid chromatography, gel electrophoresis, UV thermal melting, and circular dichroism spectroscopy. Incorporation of *closo*-dodecaborate residues at the 3′-end of all oligomers stabilized their complementary complexes, whereas analogous 5′-modification decreased duplex stability. Two boron clusters attached to the opposite ends of the oligomer only slightly influence the stability of complementary complexes of RNA oligonucleotide and its 2′-O-methyl and 2′-fluoro analogs. On the contrary, the same modification of DNA oligonucleotides significantly destabilized the DNA/DNA duplex but gave a strong stabilization of the duplex with an RNA target. According to circular dichroism spectroscopy results, two terminal *closo*-dodecaborate residues cause a prominent structural rearrangement of complementary complexes with a substantial shift from the B-form to the A-form of the double helix. The revealed changes of key characteristics of oligonucleotides caused by incorporation of terminal boron clusters, such as the increase of hydrophobicity, change of duplex stability, and prominent structural changes for DNA conjugates, should be taken into account for the development of antisense oligonucleotides, siRNAs, or aptamers bearing boron clusters. These features may also be used for engineering of developing NA constructs with pre-defined properties.

## 1. Introduction

Oligonucleotide conjugates with boron clusters attract particular attention as tools for molecular biology, biotechnology, and biomedicine. Their fields of application include potential agents for boron neutron capture therapy (BNCT) (see, e.g., [1]), diagnostic tools and probes for molecular biology [2], siRNA components [3] and antisense agents [4,5,6], and a basis for the development of nanoconstructs and novel materials for modern nanotechnologies [6,7]. The hydrophobicity of boron clusters could improve the cell penetration of oligonucleotide constructs, including siRNA and antisense oligonucleotides. Each cluster has 12 boron atoms attached to the oligonucleotides, prompting suggestions that such constructs should become effective BNCT agents for tumor treatment. Oligonucleotide-based hybridization probes containing boron clusters show promise for the electrochemical detection of nucleic acids due to their unique redox properties provided either by the clusters themselves or by their metallocomplexes [8]. Moreover, the possibility of multiple functionalization of boron clusters allows using them for creating branches in the design of oligonucleotide-based nanoconstructs.

The most common methods of incorporating boron clusters to the oligonucleotides rely on modifications of ribose 2′-position or heterocyclic bases, using pre-synthetic or post-synthetic techniques [1,9,10]. So far, only one study has been published on the terminal oligonucleotide modifications by boron clusters [11]. Along with that, most of the works on oligonucleotides bearing boron clusters deal with non-modified DNA oligonucleotides [4,5,6], with one exception for siRNA [3]. In the present work, we propose a method employing click chemistry for synthesizing novel DNA, RNA, and 2′-modified oligoribonucleotides conjugated with boron clusters as terminal 5′-, 3′- or dual modifications. Since bulky *closo*-dodecaborate residue could affect nucleic acids’ (NA) properties and disrupt their secondary structure, one should consider this possibility when incorporating boron clusters into functional nucleic acids, siRNA, or antisense oligonucleotides, or when engineering nanoconstructs using oligonucleotide-boron conjugates. Therefore, we performed a systematic study of the influence of *closo*-dodecaborates residues at the terminal positions of oligonucleotides on their essential characteristics, particularly on the properties of their complementary complexes. In this context, we compared the properties of non-modified DNA and RNA and oligonucleotides with widely used 2′-O-methyl and 2′-fluoro modifications. The results of the studies on the influence of boron clusters on the properties of oligonucleotides of different nature will be of use for choosing the optimal location for the terminal modification.

## 2. Results and Discussion

### 2.1. Synthesis of Oligonucleotide Conjugates with Boron Clusters

To attach boron clusters to oligonucleotides, we have chosen a versatile post-synthetic approach based on the Cu(I)-catalyzed [3+2] azide-alkyne Huisgen cycloaddition (CuAAC) reaction, one of the most robust bio-orthogonal methods of click chemistry for conjugating biomolecules [12,13,14]. The reaction requires an azido group in one component and an alkyne group in the other; the resulting triazole unit is very stable in a wide range of conditions.

#### 2.1.1. Synthesis of Azido Derivatives of Closo-dodecaborate

**Figure 1:**
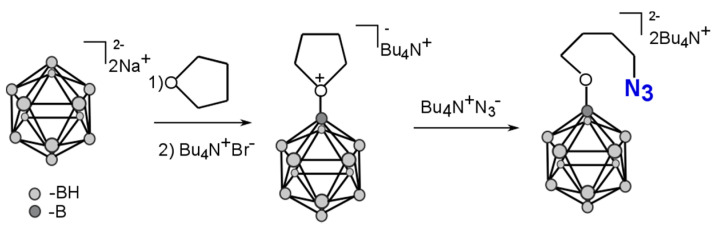
Scheme of the synthesis of the bis-tetrabutylammonium-(4-azidobuthoxy)-undecahydro-*closo*-dodecaborate (azido derivative of *closo*-dodecaborate).

An azido component of the click reaction, the derivative of *closo*-dodecaborate, was synthesized using the methods described in [15,16] with some of our modifications (Figure 1).

*Closo*-dodecaborate reacted with tetrahydrofurane in the presence of BF_3_∙Et_2_O, followed by the treatment with tetrabutylammonium azide. The obtained derivative was characterized by ^1^H-NMR and IR spectroscopy. In particular, we observed characteristic bands at 2480 cm^−1^ (B-H bond) and 2100 cm^−1^ (-N_3_ group) in the IR spectrum (Figure S1).

#### 2.1.2. Synthesis of 5′- and/or 3′-Alkyne-Modified Oligonucleotides

The synthesis of 3′-alkyne derivatives of oligonucleotides was performed using modified polymer support 3′-alkyne-Modifier Serinol controlled pore glass (CPG) for solid-phase phosphoramidite synthesis. For 5′-alkyne derivatives, we employed the method previously described by us [11] based on the activation of a 5′-hydroxy group of protected support-bound oligonucleotide by *N,N′*-disuccinimidyl carbonate (DSC) followed by the interaction with propargylamine. The series of alkyne-modified oligomers included 3′-, 5′- and dual 3′,5′-modified oligodeoxyribonucleotides, oligoribonucleotides, oligo(2′-O-methylribonucleotides), and oligoribonucleotides with all pyrimidine nucleotides replaced by their 2′-fluoro analogs.

#### 2.1.3. Conjugation of Closo-dodecaborates to 5′- and/or 3′-Alkyne Modified Oligonucleotides

**Figure 2:**
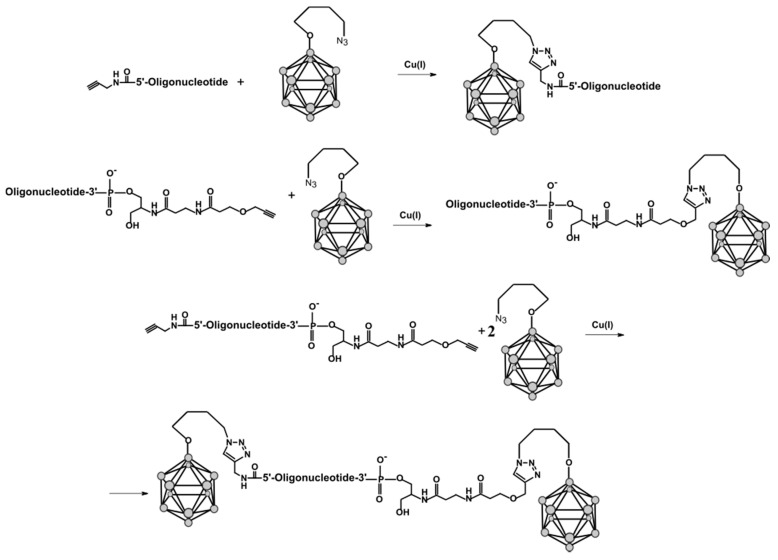
Schemes of the incorporation of *closo*-dodecaborate azido derivatives into 5′-, 3′- or dual 5′,3′-alkyne-modified oligonucleotides using click chemistry.

The click reaction of alkyne-modified oligonucleotides with an azido derivative of *closo*-dodecaborate was carried out according to the reaction schemes shown in Figure 2. The structures and characteristics of synthesized conjugates of DNA (**D**), RNA (**R**), 2′-O-methyl RNA (**M**), and 2′-fluoro-pyrimidine RNA (**RF**) oligonucleotides are given in Table 1.

The proposed post-synthetic approach excludes the treatment of the boron cluster by amines and fluoride ions, which are routinely used for oligonucleotide deprotection, thus minimizing the possibility of side reactions of *closo*-dodecaborate. After click reaction, the conjugates were isolated by preparative polyacrylamide gel electrophoresis (PAGE) followed by desalting and characterized by the MALDI-TOF mass spectrometry (Table 1).

### 2.2. Physico-Chemical Properties of the Oligonucleotide Conjugates with Closo-Dodecaborates

#### 2.2.1. RP-HPLC and Electrophoretic Analysis of the Conjugates

**Figure 3:**
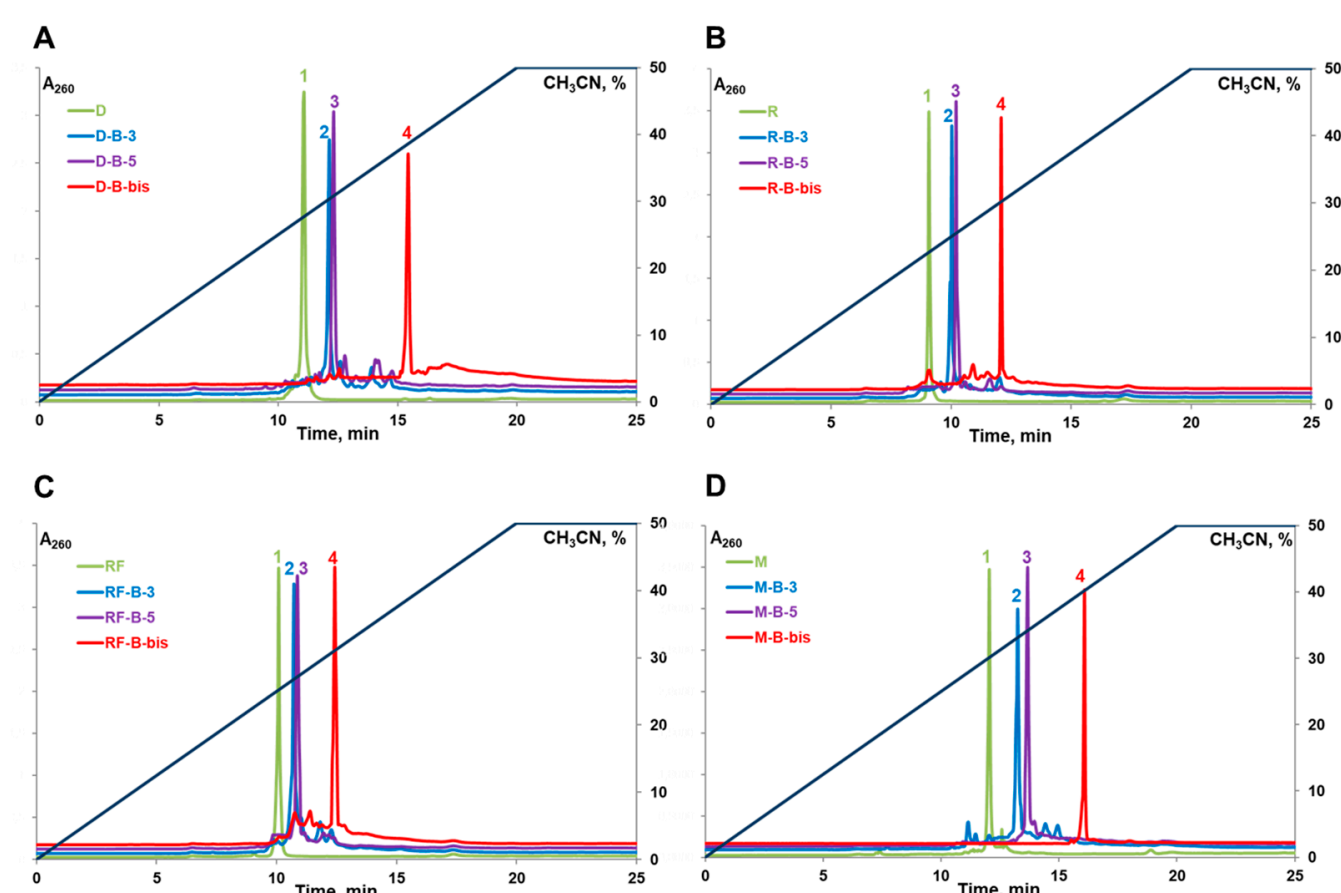
Reversed-phase high-performance liquid chromatography (RP-HPLC) analyses of parent oligonucleotides (profile 1) and their 3′-conjugates (profile 2), 5′-conjugates (profile 3), and dual 3′,5′-conjugates (profile 4) with *closo*-dodecaborate. (**A**)—oligodeoxyribonucleotide **D** and its conjugates; (**B**)—oligoribonucleotide **R** and its conjugates; (**C**)—2′-F-Py RNA oligomer **RF** and its conjugates; (**D**)—oligo(2′-O-methylribonucleotide) **M** and its conjugates. See Materials and Methods for the RP-HPLC conditions.

The analysis of synthesized oligonucleotide conjugates by reversed-phase high-performance liquid chromatography (RP HPLC) showed an expected increase in their retention times provided by boron clusters. The retention times also depended on the nature of the oligonucleotide component and increased in the row RNA < 2′-F-RNA < DNA < 2′-O-Me-RNA (Figure 3, Table 1). Conjugates with boron clusters also demonstrated decreased electrophoretic mobility during denaturing PAGE compared to parent oligonucleotides (Figure S2). Therefore, both chromatographic and electrophoretic assays showed that incorporation of one boron cluster to the 3′- or 5′-end of oligonucleotide increases its hydrophobicity, and this effect takes place for all types of oligonucleotides under investigation. Two *closo*-dodecaborate residues attached to both termini of the oligonucleotides further enhanced their hydrophobicities.

#### 2.2.2. Thermal Denaturation Studies of the Boron Clusters Modified Duplexes

**Figure 4:**
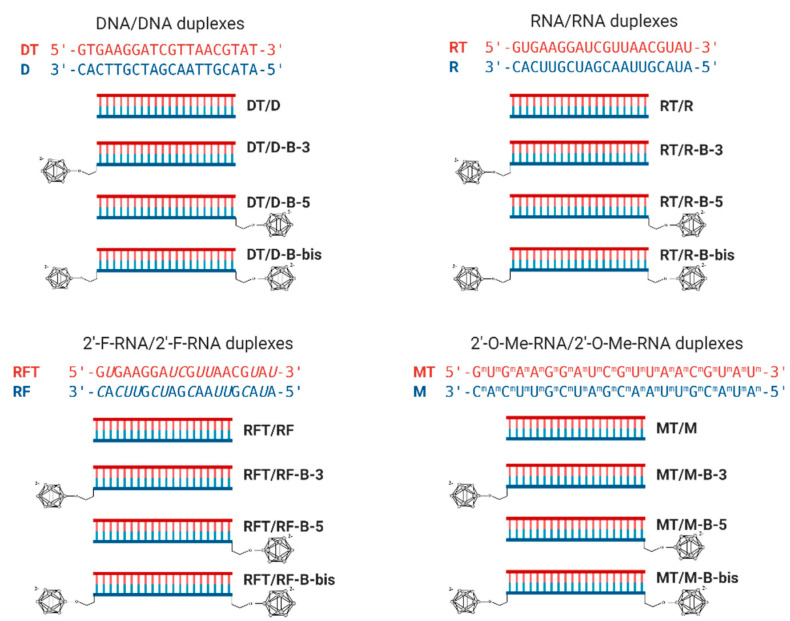
Complementary complexes of oligonucleotides bearing of *closo*-dodecaborate at 5′- and/or 3′-terminus of one of the components.

To evaluate the influence of the terminally attached *closo*-dodecaborates on the duplex stability, we examined melting profiles for the duplexes formed by oligonucleotides and their conjugates with complementary DNA (**DT**), RNA (**RT**), 2′-F-RNA (**RFT**), or 2′-O-Me-RNA (**MT**) strands by UV-melting experiments at 260 and 270 nm. The nucleotide sequences of both strands and structures of the duplexes are presented in Figure 4.

**Figure 5:**
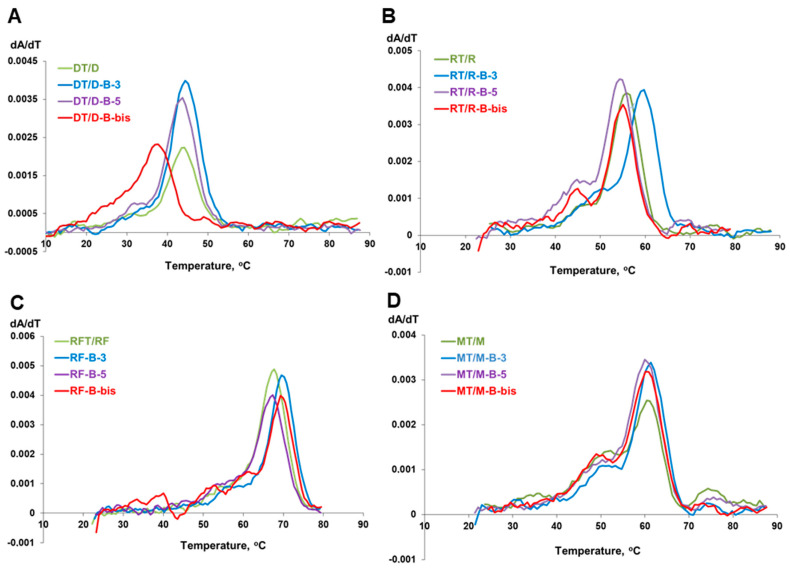
Melting curves of the duplexes formed by oligonucleotide conjugates with *closo*-dodecaborates and their complements: DNA (**A**), RNA (**B**), 2′-F-RNA (**C**), and 2′-O-Me-RNA (**D**). For each panel, green curve—control duplex of parent non-conjugated oligonucleotide, blue curve—duplex of 3′-conjugate, violet curve—duplex of 5′-conjugate, red curve—duplex of dual 3′,5′-conjugate. Conditions: 10 mM sodium phosphate (pH 7.5), 2 µM oligonucleotides.

**Figure 6:**
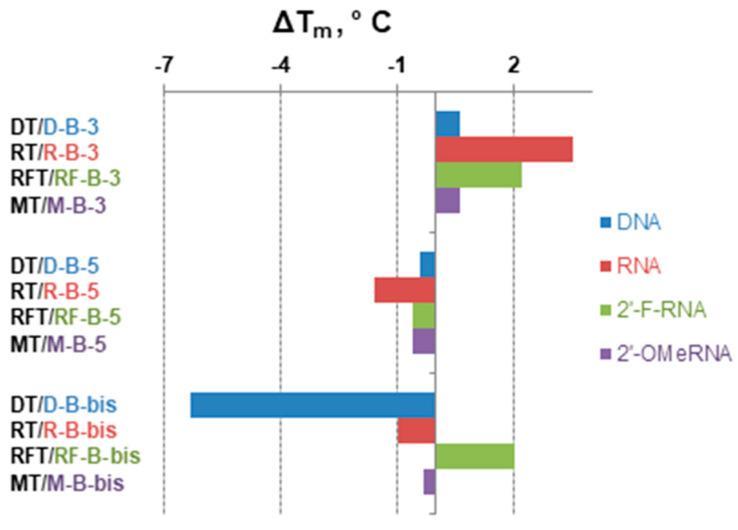
The differences between T_m_ values for homoduplexes formed by non-modified parent oligonucleotides and by corresponding *closo*-dodecaborate conjugates.

The thermal melting of all duplexes exhibited sigmoidal melting curves; their first derivatives are presented in Figure 5. The melting temperatures were determined as maximums of differentiated melting curves (Figure 5, Table 2). The analysis of the results revealed that incorporating the *closo*-dodecaborate at the 3′-end of the oligonucleotides increases the melting temperature with complementary oligonucleotide for 0.6–3.5 °C (Figure 6). By contrast, analogous 5′-modification in all cases decreased the duplex stability for 0.4–1.6 °C. These differences may be attributed to the distinctive conformational features of modifying units located at opposite ends of the oligonucleotide chain, i.e., boron clusters together with the 3′-linker or 5′-linker, which have different structures (Figure 2).

A simultaneous modification of both ends of oligoribonucleotide (**R-B-bis**) and oligo(2′-O-methylribonucleotide) (**M-B-bis**) decreased the stability of their complementary complexes. The duplex of the dual conjugate of DNA oligonucleotide (**D-B-bis**) demonstrated the most prominent decrease of stability with a melting temperature 6.3 °C lower than the corresponding control duplex. Only for the duplex of 2′-fluoro modified RNA bearing two boron clusters (**RF-B-bis**), resulted in a 2 °C increase of the melting temperature. A significant duplex destabilization for the dual conjugate of the DNA oligonucleotide should most probably originate from the disturbance of the structure of the complex and change of the form of the double helix after incorporating two boron clusters. To verify this suggestion, we performed CD spectroscopy studies (see below).

**Figure 7:**
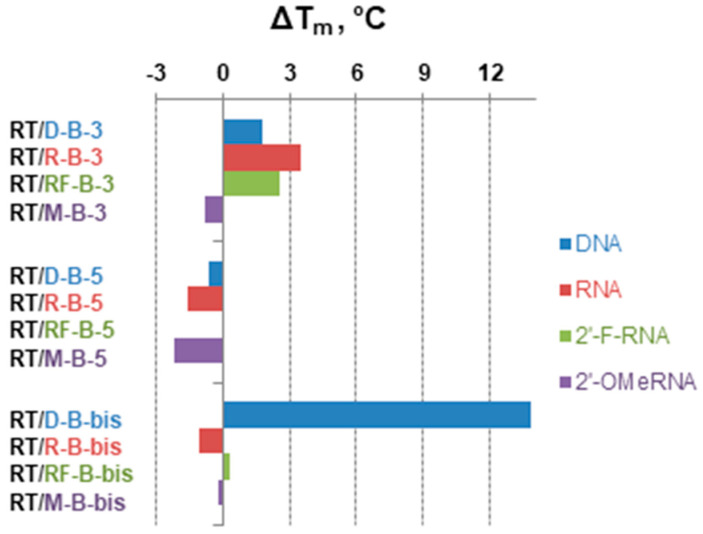
The differences between T_m_ values for duplexes formed by non-modified parent oligonucleotides and by corresponding *closo*-dodecaborate conjugates with RNA target **RT**.

Oligonucleotides and their modified analogs are widely used as a basis for the development of antisense agents for targeted gene therapy of different diseases [17,18,19]. From this point of view, it was interesting to examine the ability of oligonucleotides with terminal boron clusters to form complementary complexes with the RNA target (**RT**). We, therefore, carried out thermal melting studies for the hybrid duplexes of RNA with DNA (**D**), 2′-O-methyl RNA (**M**), and 2′-fluoro modified RNA (**RF**) oligomers and their conjugates with *closo*-dodecaborates (Table 2, Figure 7).

The duplex of 2′-fluoro modified RNA oligomer with RNA target showed the maximal stability while DNA/RNA duplex was less stable within the series. In most cases, incorporation of *closo*-dodecaborate at the 3′-end of the oligomers increased the melting temperature for 1.8–3.5 °C, with only one exception for 3′-conjugate of 2′-O-Me RNA (**M-B-3**), which showed a 0.8 °C decrease of the T_m_ value compared to the parent oligomer. Analogous 5′-modification by the boron cluster destabilized the duplexes of DNA, RNA, and 2′-O-Me RNA oligonucleotides with an RNA target for 0.6–2.2 °C, but had almost no influence (only a slight increase of 0.1 °C) on the duplex of 2-fluoro modified oligonucleotide 5′-conjugate (**RF-B-5**).

The most heterogeneous impact on the duplex stability with RNA target was observed in the case of simultaneous attachment of boron clusters to the 3′- and 5′-ends of the oligonucleotides. For the 2′-O-Me RNA oligonucleotide, dual modification (**M-B-bis**) destabilized its duplex with an RNA target. By contrast, the dual conjugate of the DNA oligomer (**D-B-bis**) demonstrated a sharp increase of duplex stability with a melting temperature 13.8 °C higher compared to the parent oligomer (**D**). For the 2′-fluoro modified RNA oligomer, dual terminal modification by the boron clusters (**RF-B-bis**) gave only a slight increase (0.3 °C) of the T_m_ value. We consider the prominent increase of stability for the RNA/DNA heteroduplex **RT/D-B-bis** as a consequence of changes in the form of the double helix. A more in-depth examination of this phenomenon is described in the next section.

#### 2.2.3. Circular Dichroism Studies of Oligonucleotides and Their Duplexes

For a deeper insight into the nature of duplex stability variations, we monitored the structural changes in NA complexes caused by the introduction of terminal *closo*-dodecaborates using circular dichroism (CD) spectroscopy [20,21,22]. This method permits to perform measurements in small amounts of material in physiological buffers and to reveal structural changes caused by additional ligands or groups or changes in environmental conditions, such as pH, temperature, and ionic strength.

Circular dichroism (CD) spectra were registered for all boron cluster-modified DNA and RNA, and their duplexes with complementary strands (Figure 7). Interestingly, the CD-spectra of complementary strands **DT** and **RT** (for their structures, see Table S1) differed from spectra of addressing oligonucleotides **D** and **R** by the intensities of characteristic bands. Most probably, this effect arises from different nucleotide sequences [20]. A similar phenomenon was observed for 2′-F-RNA and 2′-O-Me-RNA oligonucleotides (Figure S3).

**Figure 8:**
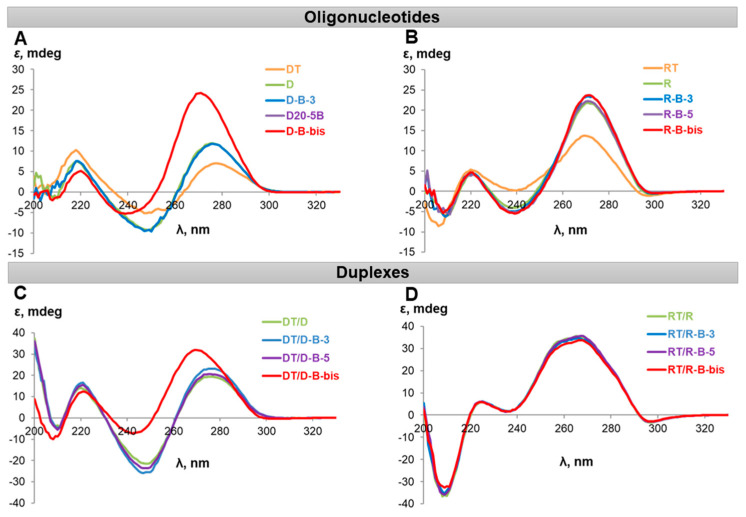
Circular dichroism (CD) spectra of boron cluster-modified oligonucleotides (**A**,**B**) and their duplexes (**C**,**D**) with complementary strands. (**A**) Spectra of oligodeoxyribonucleotides; (**B**) spectra of oligoribonucleotides; (**C**) spectra of DNA duplexes; (**D**) spectra of RNA duplexes. Conditions: 25 °C, 10 mM sodium phosphate (pH 7.5), 100 µM oligonucleotides.

In general, incorporation of boron clusters did not significantly change the profile of CD spectra for homoduplexes, with only one exception. The simultaneous modification of 3′- and 5′-ends of oligodeoxyribonucleotide by boron clusters (**D-B-bis**) significantly changed the spectrum shape of DNA/DNA duplexes, making it similar to the RNA/DNA spectrum. CD spectra of the duplexes formed by two complementary strands exhibited the curves typical for the B-form helix geometry in the case of DNA (Figure 8C) or for the A-form helix geometry in the cases of RNA, 2′-F-RNA, and 2′-O-Me-RNA (Figure 8D and Figure S3C,D) [20]. Quite surprisingly, the DNA duplex containing two *closo*-dodecaborate residues demonstrated the CD spectrum intermediate between the B- and A-forms of duplexes. We observed a shift of a 275 nm positive peak to 265 nm, a decrease of intensity of the negative peak at 245 nm, and also a slight decrease of intensity of the negative peak at 210 nm.

**Figure 9:**
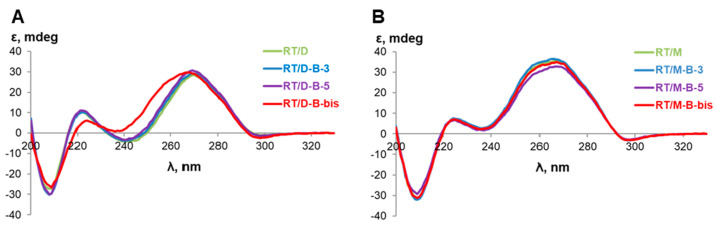
Circular dichroism (CD) spectroscopy curves of heteroduplexes formed by boron cluster-modified oligonucleotides with RNA-target (**RT**). (**A**) RNA/DNA duplexes; (**B**) RNA/2′-O-Me-RNA duplexes. Conditions: 25 °C, 10 mM sodium phosphate (pH 7.5), 100 µM oligonucleotides.

In the case of heteroduplexes with the RNA target **RT**, CD spectra of all duplexes formed by DNA, 2′-O-Me RNA, and their conjugates with boron clusters showed profiles clearly pointing to the A-form of the duplex. An example of typical CD profiles is given in Figure 9B.

Most interestingly, CD spectra of hybrid duplexes formed by the RNA target and DNA oligonucleotide or its mono-conjugates with *closo*-dodecaborate showed profiles typical for the family of the A-form double helix. Namely, the positive peak around 275 nm moved to 265 nm, while the intensity of the negative peak around 245 nm decreased, and the intensity of the negative band around 210 nm significantly increased. Moreover, the profile of the CD spectrum for the duplex of RNA target with dual DNA conjugate **D-B-bis** bearing two boron clusters almost completely shifted to the A-form of the double helix.

Therefore, incorporating boron clusters to the terminal positions of RNA oligonucleotide and its 2′-modified analogs (2′-O-methyl and 2′-fluoro) only slightly influenced the structure of oligonucleotides and their duplexes. Simultaneously, the same terminal modifications of DNA oligonucleotides had a pronounced effect on the oligomer itself and its duplexes and provided a decisive shift to the A-form of the double helix, most prominently for the hybrid RNA/DNA duplex.

The revealed changes of characteristics for oligonucleotides of different types appearing after incorporating terminal boron clusters, such as increased hydrophobicity, change of duplex stability, and prominent structural changes for DNA conjugates, should be taken into account for the development of antisense oligonucleotides, siRNAs, or aptamers bearing boron clusters. These features may also be used for engineering of developing NA constructs with pre-defined properties.

## 3. Materials and Methods

### 3.1. Chemicals and Reagents

A controlled pore glass support (CPG) derivatized with 2′-*O*-TBDMS-C, 2′-*O*-TBDMS-U, 2′-*O*-methyl-C, 2′-*O*-methyl-U,deoxycytosine (dC) or deoxythymidine (dT), 5′,*N*-protected 2′-*O*-methylribo- (A, C, G or U), 2′-*O*-TBDMS-ribo (A, C, G or U) and deoxyribo (dA, dC, dG or dT), phosphoramidites, and 3′-alkyne-Modifier Serinol CPG were purchased from Glen Research Inc. (Sterling, VA, USA), and 5′,*N*-protected 2′-deoxy-2′-fluoro purine phosphoramidites and CPG were purchased from ChemGene Corp (Wilmington, MA, USA). *N,N*-Diisopropylethylamine (DIPEA) and propargylamine were purchased from Sigma-Aldrich (St. Louis, MO, USA), *N*,*N*′-disuccinimidyl carbonate (DSC) was purchased from Acros Organics (Geel, Belgium), 10 mM Cu(II)-TBTA Stock in 55% dimethylsulfoxide (DMSO) and ascorbic acid were purchased from Lumiprobe (Russia), and sodium dodecaborate Na_2_[B_12_H_12_] was purchased from AviaBor (Dzerzhinsk, Russia). All solvents (tetrahydrofuran (THF), DMSO, CH_3_CN (various vendors)) were dried with 3 Å molecular sieves or by distillation and stored over CaH_2_. Kieselgel F254 thin-layer chromatography plates were purchased from Merck (Kenilworth, NJ, USA).

### 3.2. Physical Measurements

1H-NMR spectra of the compounds were measured with CDCl_3_ as a solvent, using an AVANCE III 400 NMR spectrometer (Bruker Corporation, Billerica, MA, USA).

Mass spectra were recorded using a MALDI-TOF Autoflex Speed mass spectrometer (Bruker Daltonics, Billerica, MA, USA). The IR-spectrum was registered using Fourier Transform Infra Red Spectrometer 640-IR (Varian, Atlanta, GA, USA).

The optical densities of the solutions of oligonucleotides and their conjugates were measured using a NanoDrop 1000 spectrophotometer (Thermo Fisher Scientific, Waltham, MA, USA). To determine molar concentrations of the oligonucleotides and their conjugates, we used corresponding molar extinction coefficients at 260 nm calculated using the IDT OligoAnalyzer™ Tool. Molar extinction coefficients for 2′-O-Me RNA and 2′-fluoro modified RNA were taken equal to those for non-modified RNA.

### 3.3. Bis-tetrabutylammonium-(4-azidobuthoxy)-undecahydro-closo-dodecaborate (**closoB12**-azide)

Bis-tetrabutylammonium-(4-azidobuthoxy)-undecahydro-*closo*-dodecaborate was obtained according to [15,16]. A volume of 0.7 mL (0.78 g, 5.5 mmol) BF_3_xEt_2_O was added to a stirred solution of 0.95 g (5.0 mmol) Na_2_[B_12_H_12_] in 25 mL of dry THF. The reaction mixture was stirred for 12 h at room temperature, then filtered and the solvent was distilled off from the filtrate. The residue was dissolved in 70 mL water and treated with a solution of Bu_4_NBr (3.22 g, 10.0 mmol) in 30 mL water. The precipitate formed was filtered off, dried in the air, and recrystallized from methanol, giving the tetrahydrofurane-*closo*-dodecaborate complex as a crystalline product in 84% yield. ^1^H NMR (400 MHz, CDCl_3_, ppm): 4.52 (4H, dt, –O(CH_2_CH_2_)_2_), 3.21 (8H, m, Bu_4_N^+^-), 2.15 (4H, dt, –O(CH_2_CH_2_)_2_), 1.62 (8H, m, Bu_4_N^+^-), 1.44 (8H, q, Bu_4_N^+^-), 0.98 (12H, t, Bu_4_N^+^-).

The solution of Bu_4_NN_3_ (120 mg, 0.421 mmol) in CH_2_Cl_2_ (2 mL) was added to a solution of dodecahydro-*closo*-dodecaborate tetramethyleneoxonium derivative [B_12_H_11_O(CH_2_)_4_O]^−^(C_4_H_9_)_4_N^+^ (100 mg, 0.212 mmol) in 2 mL of CH_2_Cl_2_. The reaction mixture was stirred for 24 h, then washed with water (3 × 10 mL). The aqueous layer was extracted with CH_2_Cl_2_ (3 × 5 mL) and organic extracts were concentrated to dryness to obtain the desired compound (140 mg, 88%) as a colorless oil. ^1^H NMR (CDCl_3_, δ, J/Hz): 0.90 (t, 24H, CH_3_-, J = 7.1 Hz); 1.32–1.52 (m, 16H, CH_3_CH_2_-); 1.52–1.72 (m, 16H, CH_3_CH_2_CH_2_-); 3.15–3.30 (m, 18H, CH_3_CH_2_CH_2_CH_2_N-, N_3_CH_2_-); 3.55–3.67 (m, 6H, N_3_CH_2_CH_2_O-, B_12_H_11_OCH_2_CH_2_O-). IR (cm^−1^) 2465 (BH), 2105 (N_3_).

### 3.4. Synthesis of Oligonucleotides

Oligonucleotides were synthesized by the solid phase phosphoramidite method on a 0.4 µmol scale on an automated DNA/RNA synthesizer ASM-800 (Biosset, Novosibirsk, Russia) using corresponding protected phosphoramidites of 2′-O-tert-butyldimethylsilyl (2′-O-TBDMS) ribonucleotides, deoxyribonucleotides, 2′-O-methylribonucleotides, and 2′-fluoro-2′-deoxyribonucleotides (ChemGenes, USA) and protocols [23,24] optimized for the instrument. Oligonucleotides bearing a 3′-alkyne group were synthesized using a modified polymer support 3′-alkyne-Modifier Serinol CPG (Glen Research, USA). Oligonucleotides with 5′-alkyne modification were obtained in analogy with [11] (see Supplementary Materials for more details). After synthesis, cleavage from the support and deprotection of the oligonucleotides were carried out with 300 μL of 40% aq. methylamine solution at 65 °C for 15 min or 300 μL of AMA solution (ammonium hydroxide/40% aq. methylamine 1:1 v/v) at 25 °C for 2 h. The TBDMS groups of oligoribonucleotides and purine nucleotides of 2′-F-modified RNA were removed using 200 μL of mixture NMP/TEA·3HF/TEA (150/100/75) at 65 °C for 1.5 h, then treated with 300 μL of trimethylethoxysilane (TCI, Portland, OR, USA) and precipitated with diethyl ether.

### 3.5. Synthesis of Conjugates

Triethylammonium acetate buffer (pH 7.0), 10 mM *closoB12*-azide in DMSO, 5 mM ascorbic acid solution in water, and 10 mM Cu(II)-TBTA stock in 55% DMSO were added to the water solution of alkyne-modified oligonucleotide (25 nmol) according to the protocol of the click reagent supplier (Lumiprobe, Moscow, Russia). The reaction mixture was incubated at room temperature overnight. Oligonucleotide conjugates were precipitated with 2% NaClO_4_ in acetone and washed with acetone. The pellets were dried in air, dissolved in water, and analyzed by gel electrophoresis. The conversion of the oligonucleotide to the conjugate was almost quantitative, according to the polyacrylamide gel electrophoresis (PAGE) and RP HPLC data (Figure S2).

### 3.6. Purification of Oligonucleotides and Their Conjugates

Deprotected oligonucleotides and their *closo*-dodecaborate conjugates were isolated by 15% denaturing polyacrylamide gel electrophoresis (PAGE) in the 0.4 mm gel, followed by elution from the gel with 0.3 M NaClO_4_ solution, desalted with Sep-Pak C18 cartridge (Waters, USA) and precipitated as sodium salts. The purified oligonucleotide conjugates were characterized by RP-HPLC and mass-spectrometry (Table 1).

### 3.7. RP HPLC Analysis of the Oligonucleotide Conjugates

Reversed-phase HPLC (RP-HPLC) analysis of the oligonucleotides and their conjugates was performed on an Alphachrom A-02 high-performance liquid chromatograph (EcoNova, Novosibirsk, Russia) with the use of a ProntoSil-120-5-C18 AQ (75 × 2.0 mm, 5.0 μm) column, applying a gradient elution from 0% to 50% (20 min) of CH_3_CN in 0.02 M triethylammonium acetate buffer, pH 7.0 at a flow rate 100 μL per min, and detection at 260, 270, and 280 nm.

### 3.8. Melting Profiles and Thermodynamic Properties of the Duplexes Modified with Boron Clusters

All absorbance measurements were obtained in 0.2 cm pathlength cells using a Cary 300 Bio UV-VIS spectrophotometer (Varian, Palo Alto, CA, USA) equipped with a 6 × 6 thermoregulated cell holder. The duplexes of boron cluster-containing oligomers with the complementary oligonucleotides were dissolved in 10 mM sodium phosphate buffer (pH 7.5) at a final concentration of 2 μM. The melting profiles were recorded in the range 5–95 °C with a temperature gradient of 0.5 °C/min. Melting temperatures were recorded at 260 and 270 nm. The melting temperatures were calculated as a maximum of the first derivative of optical absorbance on temperature. Each result was the average from two experiments of denaturation and renaturation at two wavelengths.

### 3.9. Circular Dichroism Measurements

CD spectra of the DNA, RNA, 2′-OMeRNA, and 2′-F-RNA duplexes were recorded on a Jasco J-600 CD spectrometer (Jasco, Tokyo, Japan). The measurements were performed at room temperature in the same buffer used in the UV melting experiments (see above) at an oligonucleotide or duplex concentration of 100 μM using thermoregulated by water bath (LKB 2219 MultiTerm II, Sweden) 1 mm path length quartz cuvettes with a capacity of 180 μL, 1.0 nm bandwidth, 50 nm/min scan speed, 1 s response time, and 1 nm step resolution. Each result was the average from over 10 replicates.

## 4. Conclusions

To sum up, we attached *closo*-dodecaborates to the 3′- and/or 5′-terminal positions of oligonucleotides of different types using simple and versatile post-synthetic approaches. The series of novel mono- and bis- terminal oligonucleotide conjugates were synthesized and systematically investigated using physicochemical methods. In all cases, incorporating boron clusters increased the hydrophobicity of the conjugates compared to parent oligonucleotides. Depending on the particular location, terminally attached *closo*-dodecaborates can differently influence the stability of complementary complexes, and this feature should be accounted for in the engineering of constructs based on oligonucleotide conjugates of this type. Specifically, for DNA oligonucleotides, simultaneous incorporation of boron clusters at both 3′- and 5′-ends leads to the prominent structural change of DNA/DNA and hybrid RNA/DNA duplexes with an apparent shift to the A-form of the double helix. This feature could be useful for the engineering of antisense oligonucleotides targeted to RNA. The revealed characteristics of oligonucleotides with terminal boron clusters, particularly their thermal stability and duplex conformation, should be further considered in the development of antisense oligonucleotides, siRNAs, or aptamers bearing boron clusters, and in the design of oligonucleotide-based nanoconstructs for biomedical applications.

## Figures and Tables

**Table 1:** Oligonucleotide conjugates with *closo*-dodecaborates.

Name	Oligonucleotide Conjugate	Retention Time ^1^, min	Molecular Weight, Da ^2^	
			Found	Calcu- Lated
**D-B-3**	5′-ATACGTTAACGATCCTTCAC-**closoB12**-3′	12.1 (+1.0)	6607.78	6608.49
**D-B-5**	5′- **closoB12**-ATACGTTAACGATCCTTCAC-3′	12.3 (+1.2)	6354.15	6353.72
**D-B-bis**	5′- **closoB12**-ATACGTTAACGATCCTTCAC-**closoB12**-3′	15.3 (+4.2)	6944.07	6944.27
**M-B-3**	5′-A^m^U^m^A^m^C^m^G^m^U^m^U^m^A^m^A^m^C^m^G^m^A^m^U^m^C^m^C^m^U^m^U^m^C^m-^A^m^C^m^-**closoB12**-3′	13.2 (+1.2)	7124.05	7124.85
**M-B-5**	5′-**closoB12**-A^m^U^m^A^m^C^m^G^m^U^m^U^m^A^m^A^m^C^m^G^m^A^m^U^m^C^m^C^m^U^m^U^m^C^m^A^m^C^m^-3′	13.7 (+1.7)	6872.03	6870.08
**M-B-bis**	5′- **closoB12**-A^m^U^m^A^m^C^m^G^m^U^m^U^m^A^m^A^m^C^m^G^m^A^m^U^m^C^m^C^m^U^m^U^m^C^m^A^m^C^m^-**closoB12**-3′	16.1 (+4.1)	7460.53	7460.63
**R-B-3**	5′-AUACGUUAACGAUCCUUCAC-**closoB12**-3′	10.0 (+0.9)	6845.76	6844.32
**R-B-5**	5′- **closoB12**-AUACGUUAACGAUCCUUCAC-3′	10.2 (+1.1)	6590.84	6589.55
**R-B-bis**	5′- **closoB12**-AUACGUUAACGAUCCUUCAC-**closoB12**-3′	12.0 (+2.9)	7162.17	7162.1
**RF-B-3**	5′-AU^F^AC^F^GU^F^U^F^AAC^F^GAU^F^C^F^C^F^U^F^U^F^C^F^AC^F^-**closoB12**-3′	10.7 (+0.7)	6819.49	6818.32
**RF-B-5**	5′-**closoB12**-AU^F^AC^F^GU^F^U^F^AAC^F^GAU^F^C^F^C^F^U^F^U^F^C^F^AC^F^-3′	10.8 (+0.8)	6613.32	6613.55
**RF-B-bis**	5′-**closoB12**-AU^F^AC^F^GU^F^U^F^AAC^F^GAU^F^C^F^C^F^U^F^U^F^C^F^AC^F^-**closoB12**-3′	12.5 (+2.5)	7137.57	7136.1

^1^—Retention times upon the reversed-phase high-performance liquid chromatography (RP HPLC) (see Materials and Methods for the RP HPLC conditions). The differences from retention times for corresponding non-modified oligonucleotides (**D**, **M**, **R**, or **RF**) are given in the brackets. ^2^—Molecular mass values obtained by the MALDI-TOF mass-spectrometry. N^F^—2′-deoxy-2′-fluoro nucleotide; N^m^—2′-O-methylribonucleotide, **closoB12**—*closo*-dodecaborate introduced trough click-chemistry.

**Table 2:** The values of melting temperatures for homo- and hetero-duplexes of oligonucleotide conjugates and parent oligonucleotides.

	Homoduplexes			Heteroduplexes		
	Duplex	T_m_, °C	ΔT_m_, °C	Duplex	T_m_, °C	ΔT_m_, °C
RNA	**RT/R**	56.0				
	**RT/R-B-3**	59.5	3.5			
	**RT/R-B-5**	54.4	−1.6			
	**RT/R-B-bis**	55.0	−1.0			
DNA	**DT/D**	43.8		**RT/D**	43.8	
	**DT/D-B-3**	44.4	0.6	**RT/D-B-3**	45.6	1.8
	**DT/D-B-5**	43.4	−0.4	**RT/D-B-5**	43.2	−0.6
	**DT/D-B-bis**	37.5	−6.3	**RT/D-B-bis**	57.6	13.8
2′-F-RNA	**RFT/RF**	67.7		**RT/RF**	62.1	
	**RFT/RF-B-3**	69.9	2.2	**RT/RF-B-3**	64.7	2.6
	**RFT/RF-B-5**	67.1	−0.6	**RT/RF-B-5**	62.2	0.1
	**RFT/RF-B-bis**	69.7	2.0	**RT/RF-B-bis**	62.4	0.3
2′-O-Me RNA	**MT/M**	60.7		**RT/M**	58.6	
	**MT/M-B-3**	61.3	0.6	**RT/M-B-3**	57.8	−0.8
	**MT/M-B-5**	60.1	−0.6	**RT/M-B-5**	56.4	−2.2
	**MT/M-B-bis**	60.4	−0.3	**RT/M-B-bis**	58.4	−0.2

**ΔT_m_**—the difference between melting temperatures for complementary complex formed by parent oligonucleotide and by the corresponding *closo*-dodecaborate conjugate.

## Supplementary Materials

Supplementary Materials can be found at https://www.mdpi.com/1422-0067/22/1/182/s1. Figure S1: IR-spectra of bis-tetrabutylammonium-(4-azidobuthoxy)-undecahydro-closo-dodecaborate(closoB12-azide -azido-modified closo-dodecaborate), Figure S2: (A) Electrophoretic analysis of reaction mixtures upon synthesis of 3’ and/or 5’-conjugates of oligoribonucleotides with *closo*-dodecaborates. (B) Reversed-phase HPLC analyses of parent oligonucleotides R-Alk-3 and 3’-conjugates R-B-3. See Materials and Methods for the RP-HPLC conditions, Figure S3: Circular dichroism (CD) spectra of 2′-modified RNA oligomers bearing boron clusters (A, B), and their duplexes (C, D) with complementary strand, Table S1: Synthesized oligonucleotides.

## Abbreviations

PAGE	Polyacrylamide gel electrophoresis
CD	Circular dichroism
CPG	Controlled pore glass
DMSO	dymethylsufoxide
Tm	Melting temperature
RP-HPLC	Reversed-phase high-performance liquid chromatography
BNCT	Boron neutron capture therapy
DSC	N,N′-Disuccinimidyl carbonate
IR	Infrared spectrometry
NA	Nucleic acids
1H-NMR	Proton nuclear resonance
PAGE	Polyacrylamide gel electrophoresis
THF	Tetrahydrofuran
MALDI-TOF	Matrix-assisted laser desorption/ionization with time of flight detection
