# Supplementary material for: Terminal Mono- and Bis-Conjugates of Oligonucleotides with Closo-Dodecaborate: Synthesis and Physico-Chemical Properties
Source: Int J Mol Sci. 2020 Dec 26;22(1):182. doi: 10.3390/ijms22010182 (PMC7795522; doi:10.3390/ijms22010182)
Supplement: Supplementary file 1 [file ijms-22-00182-s001.pdf]

Supplementary Materials

Terminal mono- and bis-conjugates of oligonucleotides with *closo*-dodecaborate: synthesis and physico-chemical properties

Darya S. Novopashina [1,*], Mariya A. Vorobjeva [1], Alexander A. Lomzov [1], Vladimir N. Silnikov [1], and Alya G. Venyaminova [1]

[1] Institute of Chemical Biology and Fundamental Medicine SB RAS, Russia, Novosibirsk, Lavrentiev ave. 8
* Correspondence: danov@niboch.nsc.ru

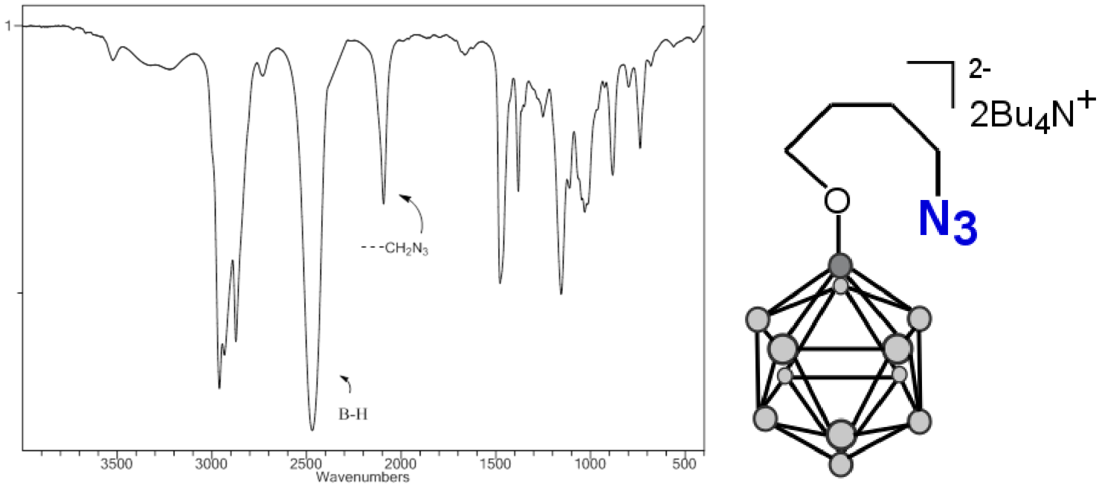

Figure S1. IR-spectra of bis-tetrabutylammonium-(4-azidobuthoxy)-undecahydro-*closo*-dodecaborate (***closoB12***-azide - azido-modified *closo*-dodecaborate).

Table S1. Synthesized oligonucleotides

**Name**	**Sequence, 5'-3'**
	**Target oligonucleotides**
**DT**	5'-GTGAAGGATCGTTAACGTAT
**MT**	5'-$G^mU^mG^mA^mA^mG^mG^mA^mU^mC^mG^mU^mU^mA^mA^mC^mG^mU^mA^mU^m$
**RT**	5'-GUGAAGGAUCGUUAACGUAU
**RFT**	5'-$GU^FGAAGGAU^FC^FGU^FU^FAAC^FGU^FAU^F$
	**Control oligonucleotides without terminal modifications**
**D**	5'-ATACGTTAACGATCCTTCAC
**M**	5'-$A^mU^mA^mC^mG^mU^mU^mA^mA^mC^mG^mA^mU^mC^mC^mU^mU^mC^mA^mC^m$
**R**	5'-AUACGUUAACGAUCCUUCAC
**RF**	5'-$AU^FAC^FGU^FU^FAAC^FGAU^FC^FC^FU^FU^FC^FAC^F$
	**3'-Alkyne-modified oligonucleotides**
**D-Alk-3**	5'-ATACGTTAACGATCCTTCAC-p-***Alk***-3'
**M-Alk-3**	5'-$A^mU^mA^mC^mG^mU^mU^mA^mA^mC^mG^mA^mU^mC^mC^mU^mU^mC^mA^mC^m$-p-***Alk***-3'
**R-Alk-3**	5'-AUACGUUAACGAUCCUUCAC-p-***Alk***-3'
**RF-Alk-3**	5'-$AU^FAC^FGU^FU^FAAC^FGAU^FC^FC^FU^FU^FC^FAC^F$-p-***Alk***-3'
	**5'-Alkyne-modified oligonucleotides**
**D-Alk-5**	5'-***Alk***-ATACGTTAACGATCCTTCAC
**M-Alk-5**	5'-***Alk***-$A^mU^mA^mC^mG^mU^mU^mA^mA^mC^mG^mA^mU^mC^mC^mU^mU^mC^mA^mC^m$
**R-Alk-5**	5'-***Alk***-AUACGUUAACGAUCCUUCAC
**RF-Alk-5**	5'-***Alk***-$AU^FAC^FGU^FU^FAAC^FGAU^FC^FC^FU^FU^FC^FAC^F$
	**3',5'-bis-Alkyne-modified oligonucleotides**
**D-Alk-bis**	5'-***Alk***-ATACGTTAACGATCCTTCAC-p-***Alk***-3'
**M-Alk-bis**	5'-***Alk***-$A^mU^mA^mC^mG^mU^mU^mA^mA^mC^mG^mA^mU^mC^mC^mU^mU^mC^mA^mC^m$-p-***Alk***-3'
**R-Alk-bis**	5'-***Alk***-AUACGUUAACGAUCCUUCAC-p-***Alk***-3'
**RF-Alk-bis**	5'-***Alk***-$AU^FAC^FGU^FU^FAAC^FGAU^FC^FC^FU^FU^FC^FAC^F$-p-***Alk***-3'

N^F – 2'-deoxy 2'-fluoro nucleotide; N^m – 2'-O-methylribonucleotide; p – phosphate; Alk-3' – -O-CH_2-CH(CH_2OH)-NH-CO-$(CH_2)_2$-NH-CO-$(CH_2)_2$-O-CH_2-C≡CH; 5'-Alk – CH≡C-CH_2-NH-CO-.

Electrophoretic analysis of oligonucleotides and their conjugates

Oligonucleotides and their conjugates were analyzed by 15% denaturing polyacrylamide gel electrophoresis (acrylamide:N,N′-methylenebisacrylamide (30:1), 8 M Urea, 50 mM Tris-H_3BO_3, pH 8.3, 0.1 mM Na_2EDTA) in 0.4 mm gel at 50 V/cm. Solutions of oligonucleotides or conjugates (approximately 250 pmol) in 1-3 μl of water were supplied with 4-5 μl of loading buffer (0.05% xylene cyanol and 0.05% bromophenol in 8 M Urea) and samples were loaded into the gel. The solution of 50 mg "Stains-all" dye in 100 mL formamide:water (1:1) was used for visualization of oligonucleotides and their derivatives. Gels were dried using GelDryer BioRad model 583 (BioRad, USA).

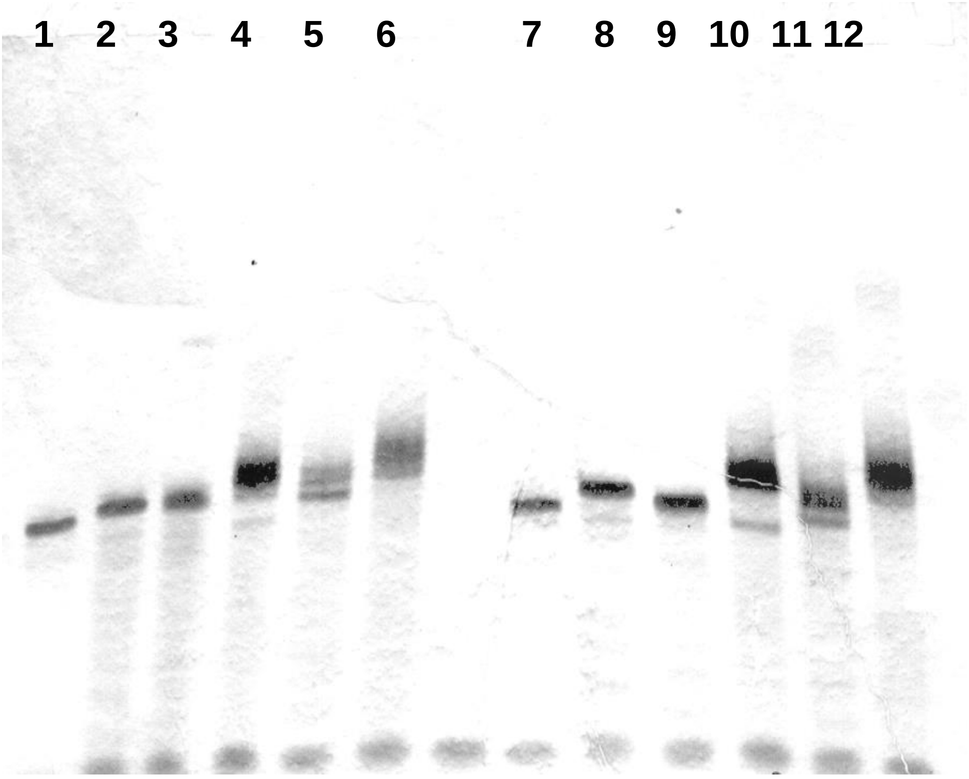

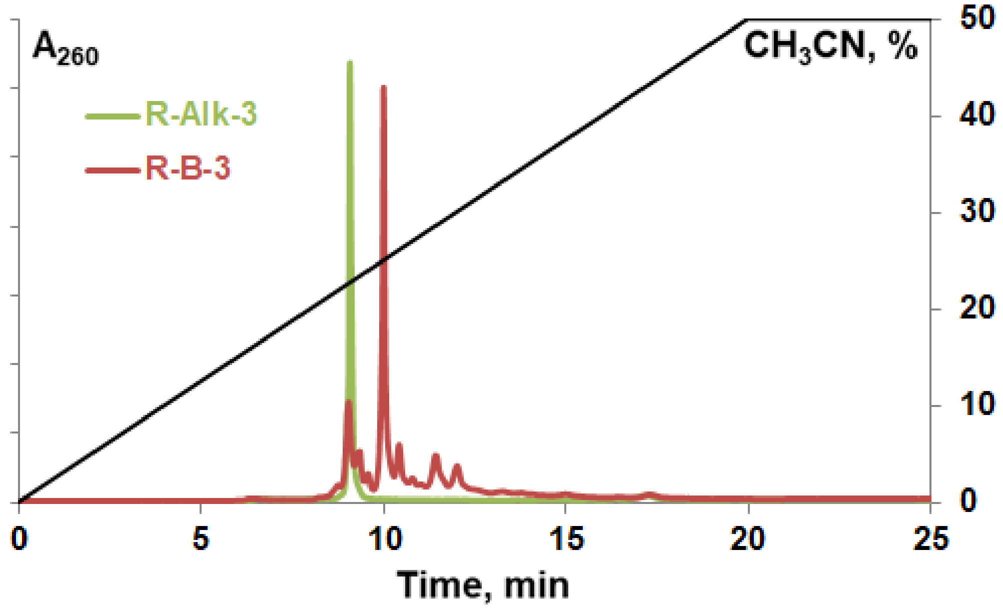

Figure S2. (**A**) Electrophoretic analysis of reaction mixtures upon synthesis of 3' and/or 5'-conjugates of oligoribonucleotides with *closo*-dodecaborates. ***Line 1*** – **R**; ***Line 2*** – **R-Alk-3**; ***Line 3*** – **R-Alk-5**; ***Line 4*** – **R-B-3**; ***Line 5***– **R-B-5**; ***Line 6*** – **R-B-bis**; ***Line 7*** – **RF**; ***Line 8*** – **RF-Alk-3**; ***Line 9*** – **RF-Alk-5**; ***Line 10*** – **RF-B-3**; ***Line 11*** – **RF-B-5**; ***Line 12*** – **RF-B-bis**. (**B**) Reversed-phase HPLC analyses of parent oligonucleotides R-Alk-3 and 3'-conjugates R-B-3. See Materials and Methods for the RP-HPLC conditions.

Solid-phase synthesis of 5′-alkyne modified oligonucleotides

N,*N′*-disuccinimidyl carbonate (6.5 mg, 25 μmol) in the mixture of acetonitrile (270 μL) and DIPEA (30 μL) was added to the dried polymer-bound DMTr-off oligonucleotide (0.25-0.5 μmol). The mixture was stirred (1500 rpm) for 1 h at 37 °C. Then the solution was replaced by freshly prepared DSC solution of the same concentration and stirred for another 30 min. The procedure was repeated twice. After that, the solution was removed, and freshly prepared solution of propargylamine (Sigma-Aldrich, St. Louis, MO, USA) (12 μmol) in 200 μL mixture of THF and DIPEA (10%, v/v) was added. The reaction mixture was stirred overnight at 37 °C. Then the solution was removed, the polymer was washed twice by a suitable solvent THF 300 μL, twice by acetone 300 μL, and dried on air.

The modified oligoribonucleotides, oligodeoxyribonucleotides and oligo(2'-O-methylribonucleotides) were cleaved from the support and deprotected by 40% methylamine in the water at 65 °C for 15 min. In the case of 2'-F-modified oligoribonucleotides, we used AMA (ammonium hydroxide/40% aq. methylamine 1:1 v/v) treatment at 25 °C for 2 h for deprotection of heterocyclic basis and phosphates, and for cleavage of oligonucleotide from support. 2'-*O*-TBDMS groups were removed from oligoribonucleotides and 2'-F-modified oligoribonucleotides by the mixture of NMP/TEA·3HF/TEA (150/100/75) at 65 °C for 1.5 h with subsequent treatment with trimethylethoxysilane (TCI, Portland, OR, USA) and precipitation by diethyl ether.

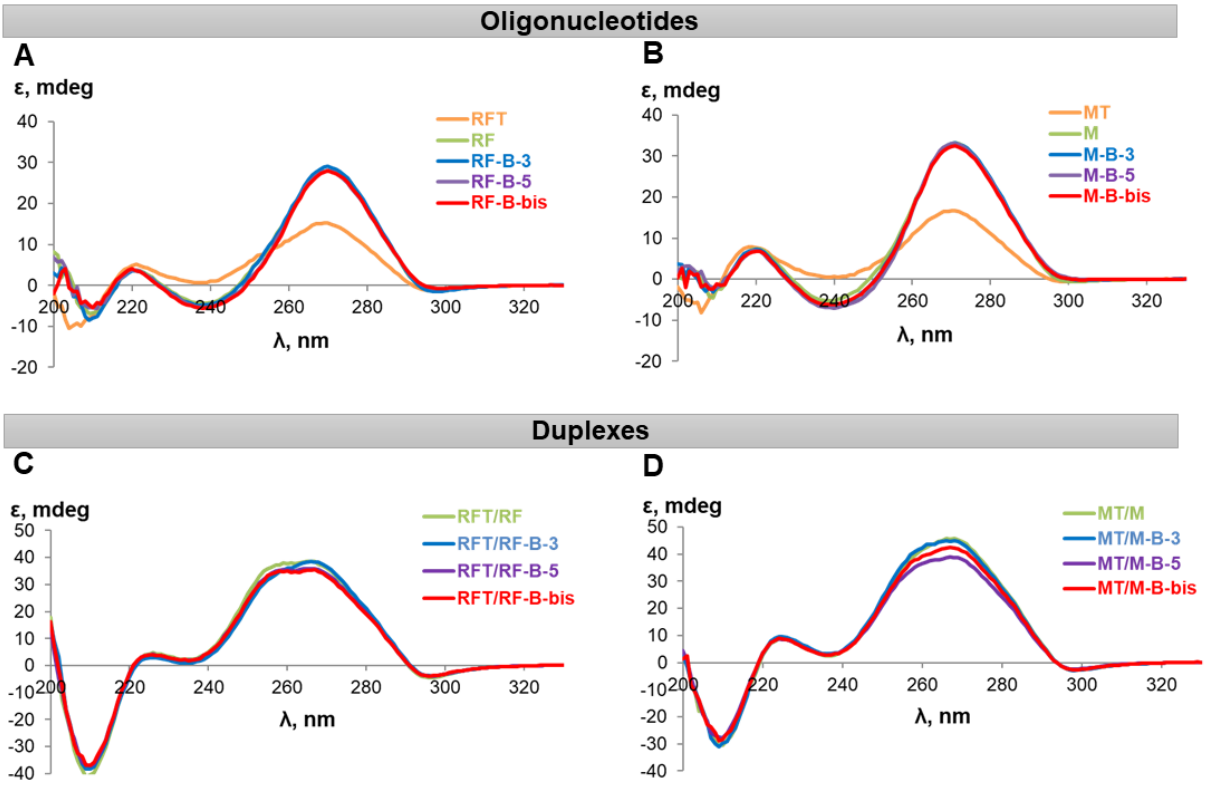

Figure S3. Circular dichroism (CD) spectra of 2′-modified RNA oligomers bearing boron clusters (A, B), and their duplexes (C, D) with complementary strand. (A) Spectra of 2′-F-Py RNA oligonucleotides; (B) spectra of 2′-O-Me RNA oligonucleotides; (C) spectra of 2′-F-Py RNA duplexes; (D) spectra of 2′-O-Me RNA duplexes. Conditions: 25 °C, 10 mM sodium phosphate (pH 7.5), 100 μM oligonucleotides.
